# Compensation for Traveling Wave Delay Through Selection of Dendritic Delays Using Spike-Timing-Dependent Plasticity in a Model of the Auditory Brainstem

**DOI:** 10.3389/fncom.2018.00036

**Published:** 2018-06-05

**Authors:** Martin J. Spencer, Hamish Meffin, Anthony N. Burkitt, David B. Grayden

**Affiliations:** ^1^NeuroEngineering Laboratory, Department of Biomedical Engineering, University of Melbourne, Melbourne, VIC, Australia; ^2^Centre for Neural Engineering, University of Melbourne, Melbourne, VIC, Australia; ^3^Victorian Research Laboratory, National ICT Australia, Sydney, NSW, Australia; ^4^National Vision Research Institute, Australian College of Optometry, Carlton, VIC, Australia; ^5^Department of Optometry and Vision Sciences, ARC Centre of Excellence for Integrative Brain Function, University of Melbourne, Melbourne, VIC, Australia

**Keywords:** octopus cells, spike-timing dependent plasticity, dendritic delay, auditory brainstem, cochlear nucleus

## Abstract

Asynchrony among synaptic inputs may prevent a neuron from responding to behaviorally relevant sensory stimuli. For example, “octopus cells” are monaural neurons in the auditory brainstem of mammals that receive input from auditory nerve fibers (ANFs) representing a broad band of sound frequencies. Octopus cells are known to respond with finely timed action potentials at the onset of sounds despite the fact that due to the traveling wave delay in the cochlea, synaptic input from the auditory nerve is temporally diffuse. This paper provides a proof of principle that the octopus cells' dendritic delay may provide compensation for this input asynchrony, and that synaptic weights may be adjusted by a spike-timing dependent plasticity (STDP) learning rule. This paper used a leaky integrate and fire model of an octopus cell modified to include a “rate threshold,” a property that is known to create the appropriate onset response in octopus cells. Repeated audio click stimuli were passed to a realistic auditory nerve model which provided the synaptic input to the octopus cell model. A genetic algorithm was used to find the parameters of the STDP learning rule that reproduced the microscopically observed synaptic connectivity. With these selected parameter values it was shown that the STDP learning rule was capable of adjusting the values of a large number of input synaptic weights, creating a configuration that compensated the traveling wave delay of the cochlea.

## Introduction

A singular sensory event such as a flash of light, the sound of a clap, or the onset of a glottal pulse in speech may create a neural response that is temporally diffuse. In the auditory domain this happens as a result of the traveling wave delay in the cochlea for example (Greenberg et al., 1997; Elberling et al., 2007; Ruggero and Temchin, 2007). This is an artifact of the frequency decomposition process and introduces a differential delay across auditory nerve fibers (ANFs) of different characteristic frequencies (CFs).

Octopus cells are located in the posteroventral cochlear nucleus (Harrison and Irving, 1966; Osen, 1969) and receive excitatory input from a relatively large number of ANFs (>60) with a broad range of CFs (Oertel et al., 2000). It has been demonstrated that octopus cells are equipped to detect and respond to coincident synaptic input (Golding et al., 1995). The combination of input asynchrony combined with their intrinsic capacity for coincidence detection appears to be paradoxical. However this incongruity is resolved if octopus cells utilize their dendritic delay to compensate for the artificial asynchrony across ANFs (McGinley et al., 2012; Spencer et al., 2012). This hypothesized configuration would require very accurately located synapses so that the post-synaptic potential's (PSP) propagation delay along the dendritic tree matches the differential delay between ANFs of differing CF. It has been noted (Golding and Oertel, 2012) that the mechanism of development and regulation of such a finely balanced synaptic configuration is yet to be understood.

The topic of this investigation, a Hebbian rule (Hebb, 1949) with a small number of assumed parameters could be capable of generating the observed CF-specific arrangement of synapses of ANFs on octopus cells. An advantage of a Hebbian rule is that it is a closed loop process; the rule adjusts the circuit's connections based on the spiking activity in the circuit itself. While an open-loop process would blindly make pre-determined connections, a closed loop rule is more rubust, and fine tuning can be achieved.

The particular rule that is hypothesized to organize the synaptic connections in this case is Spike-Timing-Dependent Plasticity (STDP) (Gerstner et al., 1996; Markram et al., 1997; Song et al., 2000). Under this rule, it is the relative timing of action potentials in the post-synaptic neuron and pre-synaptic neuron that determines the modification to each synaptic weight. If a synapse is activated immediately before the post-synaptic neuron's action potential then that synapse will be strengthened. If the reverse occurs then that synapse is weakened. This process has been observed in cortical neurons, but the existence of such a rule in the auditory brainstem is more speculative.

**Figure 1:**
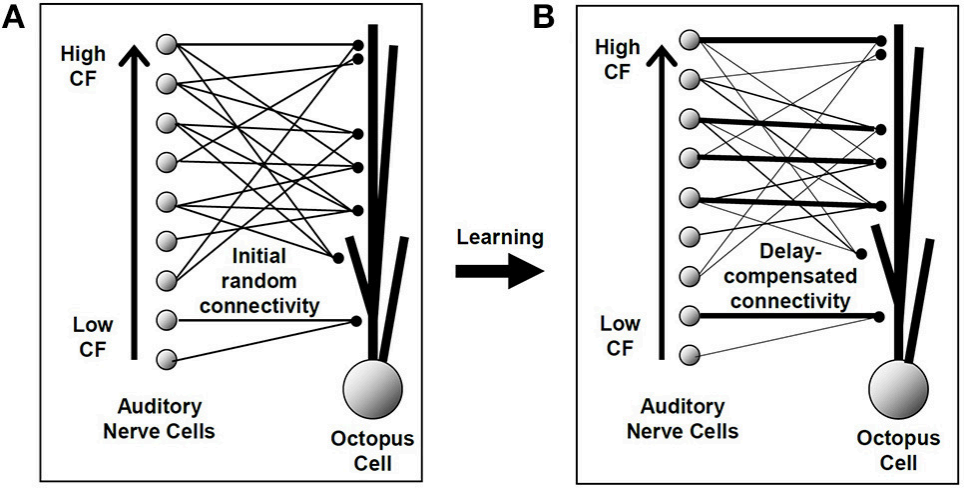
An illustration of the hypothesis to be investigated in this study. The line thickness represents the strength of the synaptic connection. **(A)** The model octopus cell was initially provided with a number of connections from each auditory nerve fiber, each assigned a random longitudinal dendritic location. **(B)** The hypothesized synaptic arrangement after the STDP learning process. Although no synapse changes position, particular synapses have been strengthened and others weakened. Under the hypothesis, this final established configuration would provide for dendritic compensation for the cochlear traveling wave delay.

Detailed electrophysiological evidence shows that in adult animals octopus cells receive ANF synaptic input in a systematic fashion along their dendrites (Willott and Bross, 1990; Oertel et al., 2000). This known configuration is shown schematically in Figure 1B. In this anatomically observed configuration the most proximal synaptic connections originate from the lowest CF ANFs (those with the longest traveling wave delay), and the most distal from the highest CF ANFs (with the shortest traveling wave delay). For the octopus cell circuit model examined in the present investigation the synaptic input was initially established randomly (Figure 1A). This assumes that initial synaptic connections are random. This is a conservative test of the hypothesis given that microscopy indicates that octopus cell dendrites are not random, but are aligned in the direction required for compensation (Oertel et al., 2000). It is hypothesized that under the action of the STDP rule synapses that do create dendritic compensation for the traveling wave delay would be strengthened by the STDP process, a process illustrated schematically in Figures 1A,B. The investigation will examine whether STDP is capable of achieving the correct synaptic configuration, and if so, to discover the “meta” parameters of the STDP rule that best achieve this.

## Methods

A model was established consisting of the following components:

- A realistic model of the auditory periphery activated by click sounds. Click sounds were chosen because these are known to be effective at activating octopus cells and they have a broadband profile.
- A modified leaky integrate-and-fire (LIF) model of the octopus cell used to recreate the firing behavior of octopus cells. This is simpler than a Hodgkin-Huxley model, but adequate to capture the relevant dynamics.
- A conventional STDP model of synaptic weight adjustments used to adjust the weights of the connections between the periphery model, and the octopus cell model.

Some parameters of the combined LIF/STDP system were selected using experimental evidence. Those parameters without sufficient basis in evidence were selected using a genetic search algorithm. The genetic algorithm was not intended to replicate any biological process. It was intended to find any set of parameters for the STDP algorithm that: (a) conformed to a physiologically plausible range of values and (b) recreated the correct known connectivity of octopus cells. These two requirements were sufficient to provide proof of principle that STDP can play the hypothesized role in octopus cells. It was not necessary to converge on a single unique set of optimal parameters. The genetic algorithm may not converge to a local or global optimum, however it was sufficient to meet the above requirements.

### ANF model and stimulus protocol

Synaptic input to the octopus cell was simulated by a realistic model of the auditory periphery, the Zilany-Bruce model (Zilany and Bruce, 2006, 2007; Zilany et al., 2009, 2014). A range of sounds, including tones, clicks, and speech sounds were used during validation of that model. The model has a middle ear filter that gives realistic responses to broadband signals. It has realistic cochlear tuning characteristics and produces appropriate jitter statistics of phase-locked spike times. It also provides physiologically realistic group delay and phase properties as a function of sound pressure level and location on the basilar membrane. In this model a sound signal is converted to action potentials in auditory nerve fibers. Each fiber responds to a different sound frequency, the fiber's CF, and each fiber possesses a different traveling wave delay corresponding to that CF.

**Figure 2:**
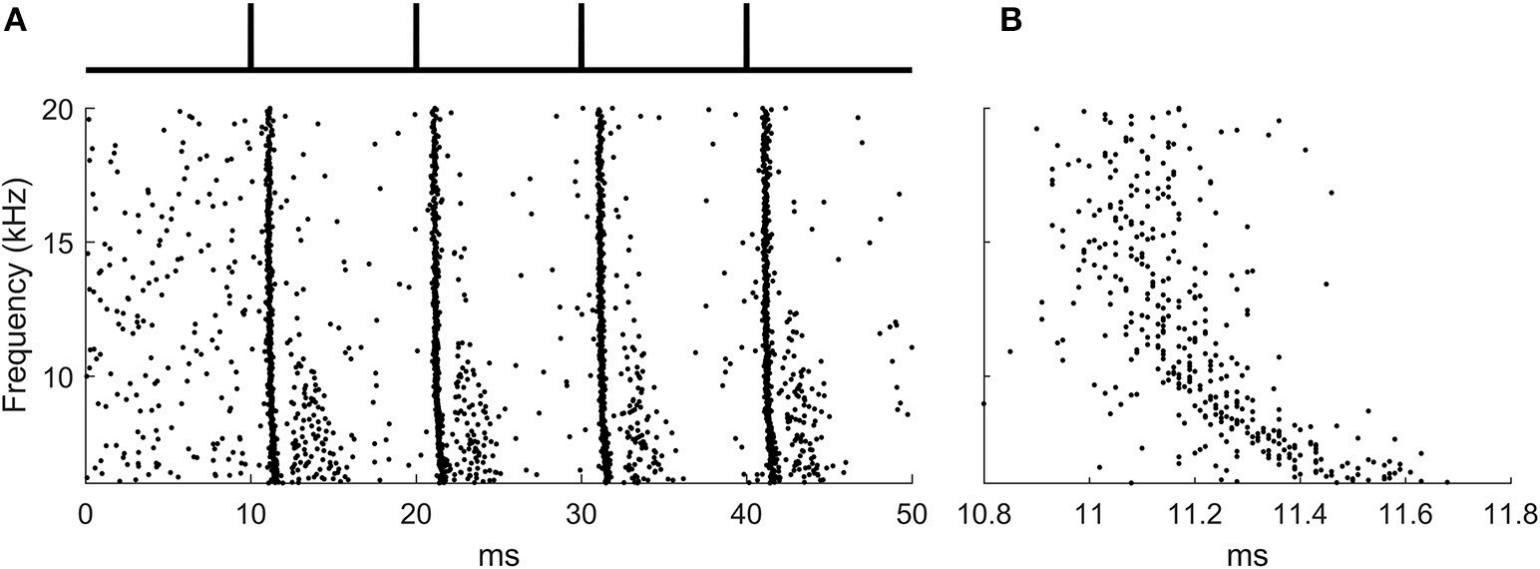
The ANF input to the octopus cell model during learning. **(A)** The raster plot produced by the auditory periphery model in response to auditory click sounds shown in schematic above. **(B)** A zoomed section of the raster plot highlighting the presence of the traveling wave delay.

The input likely to activate octopus cells are broadband clicks (Oertel et al., 2000) and this signal was used as input to the auditory periphery model (Figure 2A).

400 ANFs were modeled with each forming 3 synapses onto the octopus cell model at random locations, a total of 1,200 synapses. The stimulus lasted 50 ms, and was simulated at a time step of 10 μs. The period between ‘clicks’ in the sound stimulus was 10 ms, a time chosen for convenience and which is not critical for the function of the rule. Each learning epoch contained 4 click sounds. The frequency span of the auditory nerve fibers in the model was 6–20 kHz, chosen so that the total traveling wave delay asynchrony across the population of fibers was approximately 0.5 ms. This was to match the approximate transmission time of PSPs in octopus cell dendrites (McGinley et al., 2012; Spencer et al., 2012) (Figure 2B).

### Simplified octopus cell model

The octopus cell model is closely based on that developed in Spencer et al. (2015) for the rat. A single octopus cell was modeled using a LIF framework (Equation 1). This approach assumes that the transmembrane voltage of the octopus cell can be modeled as a single value *V*_m_ and evolves according to capacitive-resistive dynamics (Dayan and Abbott, 2005). In the conventional LIF model, when the voltage reaches some set threshold an action potential is produced, and the voltage reset. However, in the present investigation the voltage threshold is replaced by a threshold (κ) in the *rate-of-change* of the membrane potential. This was intended to recreate the known properties of the cell (Ferragamo and Oertel, 2002). When combined with input from a realistic auditory periphery this recreates the known functional properties of octopus cells (Spencer et al., 2015). The membrane voltage dynamics were described by the following equation:

(1) $$\begin{array}{l} C_{\text{m}}\frac{dV_{\text{m}}}{dt}=g_{\text{leak}}\left(V_{\text{L}}-V_{\text{m}}\right)+g_{\text{ex}}\left(E_{\text{ex}}-V_{\text{m}}\right), \end{array}$$

where *C*_*m*_ is the membrane capacitance, *g*_leak_ is the membrane's leak conductance, *V*_L_ is the reversal potential of the leak conductance, *g*_ex_ is the excitatory synapse membrane conductance, and *E*_ex_ is the excitatory reversal potential. The synaptic input was modeled using a decaying exponential function:

(2) $$\begin{array}{l} \tau_{\text{ex}}\frac{dg_{\text{ex}}}{dt}=-g_{\text{ex}}, \end{array}$$

where τ_ex_ is the time constant of decay. *g*_ex_ is incremented by an amount determined by each synapse's weight at a time determined by each synapse's position in the dendrite. The contribution from each synapse is multiplied by the value “W” which is modified by the learning homeostatic and STDP learning rules, and is initially set at 0. If the rate of change of the membrane potential exceeds 10 mV/ms (κ) over one time step then a spike is triggered and the membrane potential is reset to the leak reversal value. The value of κ was chosen to approximate the known value measured from octopus cells (Ferragamo and Oertel, 2002). There is an absolute refractory period of 1.1 ms, corresponding to a maximum firing rate of 900 Hz, the known maximum rate for octopus cells.

The dendrite of the octopus cell was modeled as a simple delay to the action potentials which were provided by the ANF model. The precise delay depends on the modeled position of the synapse on the dendrite. The maximum delay was chosen to be of the same order as that in octopus cells, 0.5 ms (McGinley et al., 2012; Spencer et al., 2012).

The values of the ANF and octopus cell model parameters are provided in Table 1.

**Table 1:** Assumed ANF and octopus cell model parameters and their values.

**Parameter**	**Value**	**References**
Membrane leak conductance (*g*_leak_)	143 nS (7 MΩ)	Oertel et al., 2000
Membrane capacitance (*C*_*m*_)	43 pF	(see caption)
Reversal potential (*V*_L_)	−65 mV	Standard value
Action potential threshold (κ)	10 mV/ms	Ferragamo and Oertel, 2002
Synapse conductance increment	1 nS	Cao and Oertel, 2010
Synapse reversal potential (*E*_ex_)	0 mV	Cao and Oertel, 2010
Maximum dendritic delay	0.5 ms	McGinley et al., 2012; Spencer et al., 2012
Number of ANF fibers	400	
Synapses per ANF fiber	3	
Total number of input synapses	1,200	

*Unreferenced values are nominal assumptions chosen at the start of the investigation and not subsequently tuned. The value of capacitance was calculated to provide a membrane time constant of 300 μs (Golding et al., 1999; Oertel et al., 2000). It is unknown how many synaptic connections are initially made to octopus cells. The value of 400 ANFs making 1,200 connections is chosen to allow adequate opportunity for learning*.

### Spike-timing-dependent plasticity and homeostasis

**Figure 3:**
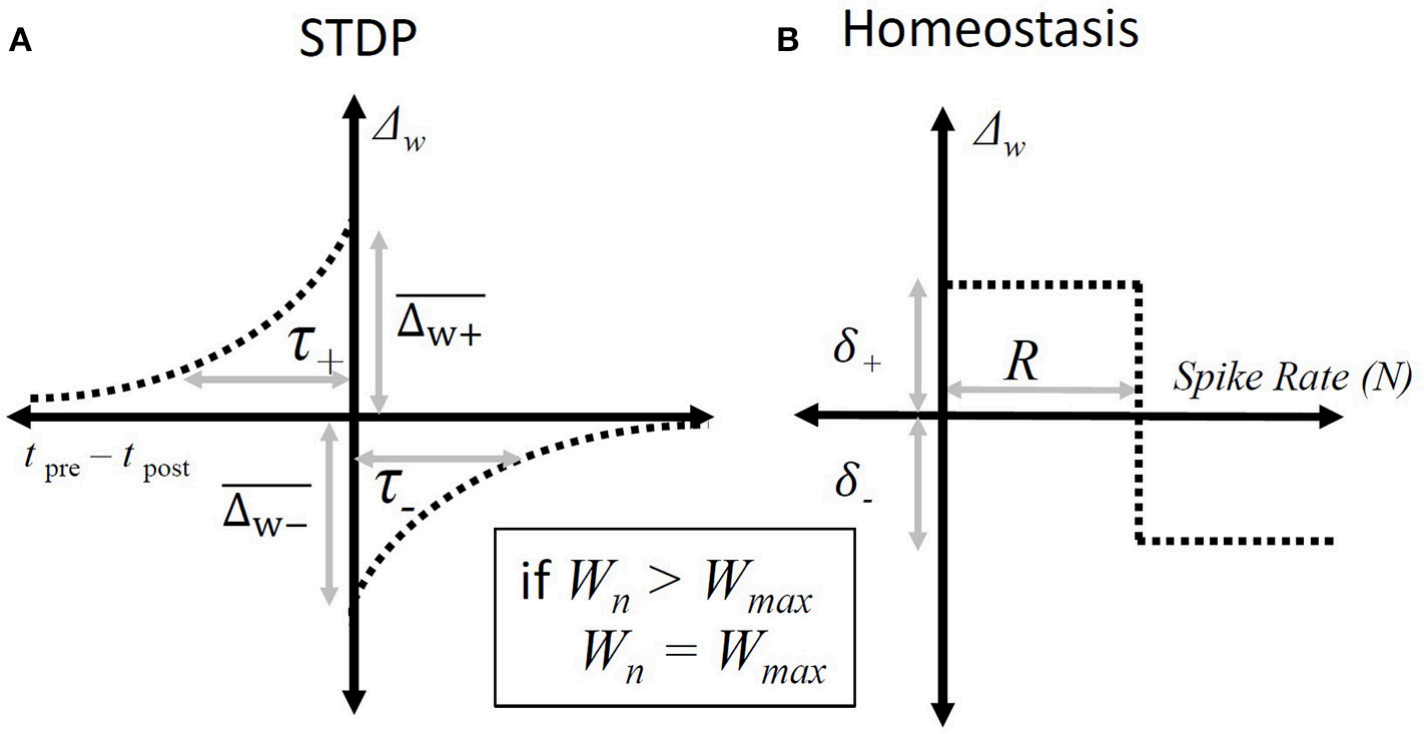
Synaptic weight adjustment. **(A)** The form of the timing dependent function, “Δ_w_”, that defines the change in a particular synapse's weight. The horizontal axis shows the relative time between any pair of presynaptic and post-synaptic spikes. The vertical axis indicates the amount by which the weight of the synapse associated with the presynaptic spike is incremented. The parameters are defined in Equation (3). **(B)** The homeostatic rule that adjusts synapses based on the overall spike-rate of the octopus cell during a particular epoch. It adjusts all input synapses equally. The horizontal axis shows the number of spikes produced in a given epoch and the vertical axis the resulting change in synaptic weights following that epoch. The parameters are defined in Equation (4).

The STDP paradigm used additive adjustment to the synaptic weights. As in the standard all-to-all STDP paradigm (Song et al., 2000); if the presynaptic spike produced by the ANF precedes a spike produced by the octopus cell then that synapse will be increased in strength by an amount that depends on the intervening duration (Figure 3), and if an input ANF produces a spike that follows one produced by the model octopus cell then that synapse was weakened.

Changes in weight of each individual synapse due to spike-timing-dependent plasticity were governed by:

(3) $$\begin{array}{l} \Delta W_{j,\text{STDP}}=\{\begin{array}{cc} \bar{\Delta_{\text{w}+}}.e^{-(t_{j,\text{pre}}-t_{\text{post}})/\tau_{-}} & :t_{j,\text{pre}}-t_{\text{post}}<0\\ -\bar{\Delta_{\text{w-}}}.e^{-(t_{j,\text{pre}}-t_{\text{post}})/\tau_{+}} & :t_{j,\text{pre}}-t_{\text{post}}>0, \end{array} \end{array}$$

where Δ*W*_*j*, STDP_ is the change in a given synaptic weight resulting from a post-synaptic action potential, $\bar{\Delta_{\text{w+}}}$ and $\bar{\Delta_{\text{w-}}}$ are the maximum synaptic weight changes of the STDP window, and τ_+_ and τ_−_ are the time constants of the STDP window, *t*_*j*, pre_ − *t*_post_ is the key parameter involved in STDP Hebbian plasticity, the time difference between the arrival of the pre-synaptic potential and the post-synaptic action potential.

In general it is known that in real cells homeostasis establishes a useful working-point for STDP to occur (Sjöström et al., 2001). A homeostatic plasticity rule was used to establish a useful working point for the activity of the STDP rule:

(4) $$\begin{array}{l} \Delta W_{j,\text{homeostasis}}=\{\begin{array}{cc} \delta_{-} & :N_{\text{post}}>R\\ \delta_{+} & :N_{\text{post}}<R, \end{array} \end{array}$$

where *N*_*post*_ is the total number of spikes produced by the cell during a single epoch (which is chosen here to have a duration of 50 ms) and R was set to 4 to match the number of expected spikes during each 50 ms epoch. The rule increments the total synaptic drive if the cell is producing spikes at a rate below the nominated rate, R, and reduces the total synaptic drive if it is above the nominated rate. Note that, in the absence of post-synaptic activity, the change in synaptic weight due to homeostasis is positive. and so initiates post-synaptic activity in the cell, which is something that is necessary for STDP.

The specific components of the homeostatic plasticity rule are not intended to represent particular physiological processes. Rather, the rule is meant to be a phenomenological recreation of the effect of homeostatic regulation of the total synaptic input to the cell.

The total learning equation combines homeostasis and STDP:

(5) $$\begin{array}{l} \Delta W_{j}=\Delta W_{j,\text{homeostasis}}\left(N_{\text{post}}\right)+\Delta W_{j,\text{STDP}}\left(t_{j,\text{pre}}-t_{\text{post}}\right), \end{array}$$

where *W* ∈ [0, *W*_*max*_]. These adjustments to synaptic weight were not made continuously during learning, but once at the end of each 50 ms epoch.

### Parameter optimization

Given that there is no experimental evidence of the particular parameter values of STDP mechanisms in octopus cells it was necessary to constrain the parameters of the STDP model by selecting values that resulted in the expected final synaptic configuration of the octopus cell. A metric was established that was used to quantify the configuration of synapses after a period of STDP learning. The metric was designed to return a high value when the dendritic delay matched and compensated for cochlear traveling wave delay.

#### Quality metric

The *basic* form of the metric was chosen to be a sum of Gaussians $\sum W_{n}e^{{\left(T-x_{n}\right)}^{2}/2\sigma^{2}}$, in which *x*_*n*_ is the sum of the traveling wave delay and the dendritic delay for each individual synapse, and *T* is a constant (0.5 ms) and is the target delay of the combined dendritic delay and traveling wave delay. So, if *x*_*n*_ = 0.5 ms then that particular synapse will contribute the maximum value to the metric. *W*_*n*_ is the weight of each synapse. The value of σ was chosen to penalize synapses for which traveling wave delay did not compensate for dendritic delay (70 μs).

With this metric, synapses that increase their value of *W*_*n*_ increase the value of the metric. However, a synapse with value of |*T* − *x*_*n*_| close to 0, leading to a high value of $e^{{\left(T-x_{n}\right)}^{2}/2\sigma^{2}}$, will increase the metric more than a synapse with a large value of |*T* − *x*_*n*_|. A large value of |*T* − *x*_*n*_| indicates that the dendritic delay is not optimally compensating for the traveling wave delay.

If total synaptic weight remained constant over time and across models, as did connectivity, then this metric would be sufficient. However this is not the case, and so it was necessary to introduce normalization to compare models with different total synaptic input and delay configurations. The normalization factor had the same Gaussian form, with the mean weight and the mean difference in the traveling wave delay and dendritic delay. This final normalized metric η was:

(6) $$\begin{array}{l} \eta =\frac{\overset{N}{\underset{n=1}{\sum }}W_{n}.e^{-{\left(T-t_{TW,n}-t_{D,n}\right)}^{2}/2\sigma^{2}}}{\overset{N}{\underset{n=1}{\sum }}W_{n}}, \end{array}$$

where *t*_*TW, n*_ is the cochlear traveling wave delay for synapse n, and *t*_*D, n*_ is the dendritic delay for synapse n.

This metric allows comparison across different epochs and models. In practice, a homogenous weight distribution, whether the synapses are strong or weak, was observed to produce a metric value of approximately 0.40. A value of η higher than this represents a synaptic configuration in which the dendritic delay is compensating for the traveling wave delay.

### Search algorithm

In order to perform a multi-dimensional search efficiently a simple genetic algorithm was used. This rule was not meant to represent any real process in the animal, but simply to discover values for the parameters of the STDP learning rule and homeostatic rule that lead to the predicted final configuration of synapses.

Initially a population of 15 models was instantiated with random parameter values and evaluated. The two which produced the highest values of η were copied without modification to the next generation. Each parameter value of a further 15 models was selected randomly from the 2 “parents.” In addition, random variation was applied to each value (see below for details). The process was repeated again with the next generation, each time following the same process. In total 100 generations were completed, each consisting of 15 models each of which were evaluated over 10 epochs of sound stimulus. Each epoch consisting of 4 click sounds over the course of 50 ms, with STDP applied at the end of each stimulus and the final value of η is recorded.

#### Random parameter variation

As described, 15 “child” models were created by selecting parameter values from one of the two “parent” models. Random variation was then imposed on each value. This was done by creating a random change from the inherited value. The variation used white noise values between 0 and 1, and compressed by: (4(*x*−0.5)^3^)+0.5 to make frequent small changes, and infrequent large changes.

## Results

### Search for the preferred parameters of the STDP learning rule

Initially all 1,200 synapses from the 400 ANFs were set to be 0 weight and the model cell did not spike at all. The “genetic” search algorithm was used to find the parameters of the homeostatic rule and the STDP rule that best achieved the observed configuration of synapses for the octopus cell. The STDP parameters selected by the search algorithm were limited to a certain physiological range (Table 2).

**Table 2:** STDP model and homeostatic rule parameter values.

**Parameter**	**Value**
STDP magnitude ($\bar{\Delta_{\text{w}}}$)	0 −10 or 20
STDP window duration (τ)	20 μs − 20 ms
Homeostatic increment/decrement (δ)	0–0.03
Weights maximum (*W*_*max*_)	0.01–0.2

*The physiological range of the STDP magnitude was either 10 or 20 (see Results for more information)*.

**Figure 4:**
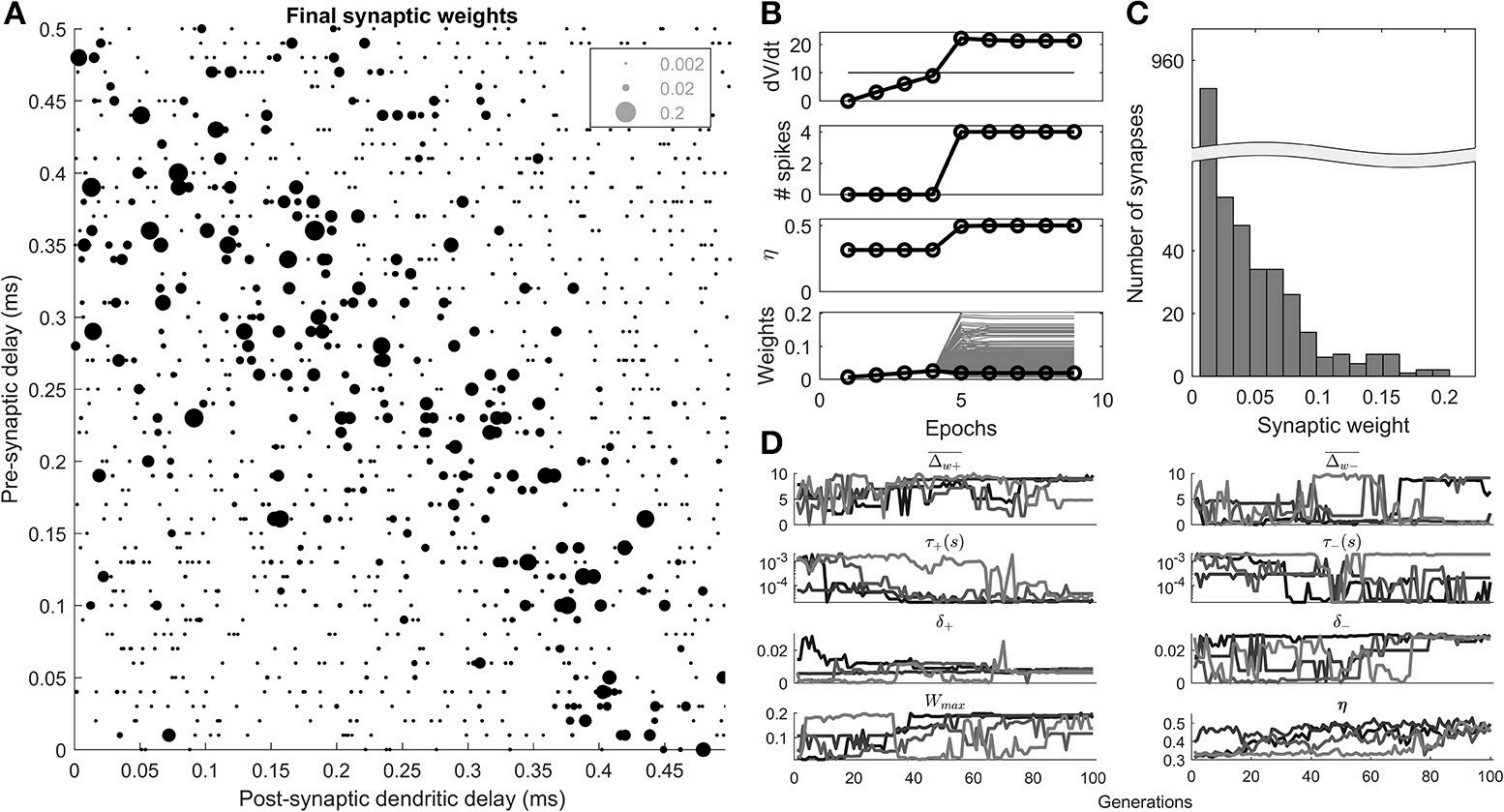
Results of the STDP learning rule, and genetic search. **(A)** An example cell's final synaptic weights shown as a function of their pre-synaptic and post-synaptic delays. **(B)** An example of the results of a 100th generation cell, during 10 epochs of learning. The maximum rate of change in voltage (*dV*/*dt*), number of spikes, η, the synaptic weights (gray), and mean synaptic weights (black) are shown as a function of 10 epochs of learning. **(C)** The distribution of the final synaptic weights. **(D)** The genetic algorithm results of 4 separate parameter explorations as a function of the search generation.

It was found that the algorithm could select synaptic weights such that the dendritic delay compensates for the traveling wave delay (Figure 4A).

A 100th generation model (Figure 4B) shows that initially (<5 epochs), the homeostatic learning rule increased the strength of the synapses uniformly resulting in higher values of *dV*/*dt* until the octopus model began to produce spikes. At the 5th epoch the STDP rule, driven by the initiation of post-synaptic spikes, came into effect and synapses were differentiated based on their contribution to the activity of the octopus cell. The final distribution of weights was unimodal (Figure 4C).

The results of 4 separate parameter searches (Figure 4D) show that all 4 searches resulted in a value of the metric (η) that is consistent with a beneficial “ordering” of the synapses along the dendrite (>0.4). This was despite the fact that some of the parameters adopted disparate values (e.g., $\bar{\Delta_{\text{w-}}}$), and others converged (e.g., τ_+_).

The “ceiling” synaptic weight parameter (*W*_*max*_) reduced to a level that did intervene in synaptic weight growth but was not highly restrictive (Figure 4D). This can be observed in the final distribution of synaptic weights (Figure 4C) which as noted, was not bimodal, something that would occur with a highly restrictive synaptic weight ceiling.

It can be seen in Figure 4D that the value of η fluctuates from generation to generation, and does not simply monotonically increase. This was because of the random element introduced by the particular synaptic connectivity instantiated from generation to generation.

The most notable feature of the parameter search was the fact that in all 4 searches the temporal width of the positive part of the STDP window, τ_+_, appeared to approach the lowest available value of 20 μs (4D). This result is explored in a further parameter exploration later in the paper.

### Octopus cell dynamics before and after synaptic weight adjustment through STDP

**Figure 5:**
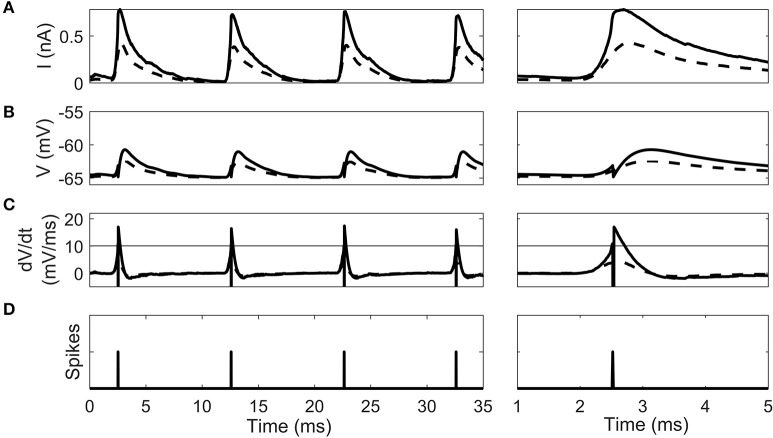
The intrinsic dynamics of the octopus cell model before STDP learning (dashed line) and after (solid line) in a 100th generation cell. Two time periods are shown. **(A)** The total synaptic current during the stimulus. **(B)** The cell voltage. **(C)** The instantaneous rate of change of the membrane voltage. The horizontal line gives the threshold of spiking. Note that the dashed pre-learning curve does not reach threshold. **(D)** The time of action potentials produced by the model after learning. Note that no output spike was produced in the pre-learning model.

The cellular dynamics of a 100th generation cell before and after 10 epochs of STDP learning show the effect of synaptic selection (Figure 5). After the 1st epoch and before STDP had any effect, the octopus cell synapses have a homogenous synaptic weight. Due to the fact that they have randomized input delays and dendritic delays this led to a synaptic current changing more slowly during a click sound than after STDP (Figure 5A). This in turn led to a smooth membrane voltage compared to after STDP learning (Figure 5B) and smooth rate of change in membrane voltage compared to after STDP learning (Figure 5C). Note that before the STDP learning process the instantaneous rate of change in the membrane voltage did not reach the spike-threshold and so did not produce a spike. After STDP learning, the synapses with a dendritic delay that compensates for input asynchrony were strengthened, and others weakened. The effect of this on the model cell's response can be seen as a sharpening of the total input synaptic current and membrane voltage (solid line in Figure 5A,B). This in turn increases the rate of change in the membrane voltage (solid line in Figure 5C) which exceeded the spike threshold and produced action potentials.

### The effect of STDP window size on the synaptic weight profile

In the previous parameter exploration (Figure 4) the value of the positive STDP window width (τ_+_) approached the value considered to be the physiological minimum (20 μs).

**Figure 6:**
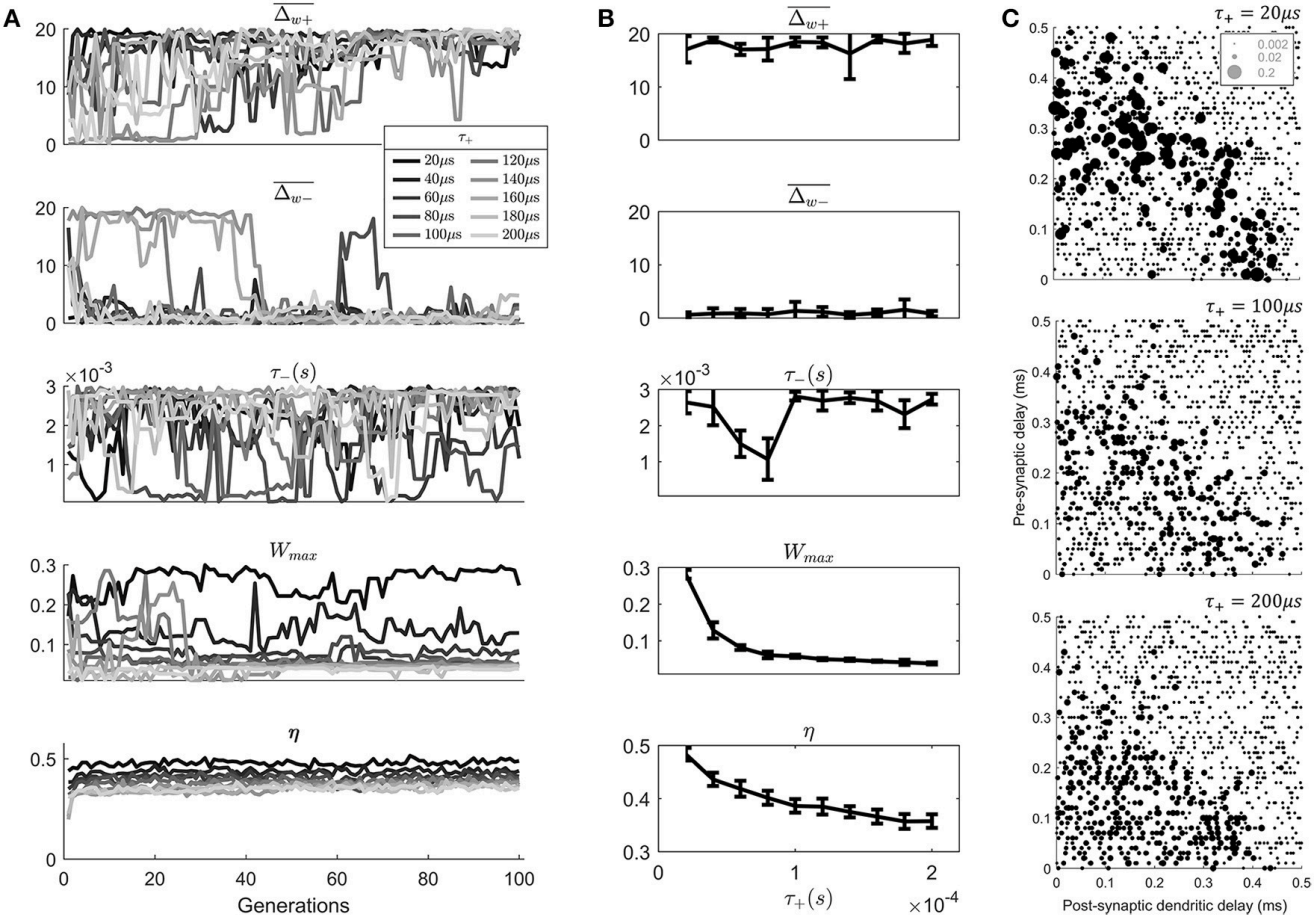
An exploration of the effect of the value of τ_+_ on the model results. **(A)** The evolution of the STDP learning rule meta-parameters and the metric η, as a function of the search generation. **(B)** The mean values of generations 80–100 of each parameter as a function of the value of τ_+_. Error bars show the standard deviation across these generations. **(C)** Final synaptic strengths associated with 3 models of STDP after varying value of τ_+_ (20, 100, 200 μs).

In order to examine the effect of different physiological minimums on the outcome of the STDP learning the value of τ_+_ was systematically varied (Figure 6). In addition, the other parameters that converged in the previous parameters exploration δ_+_ and δ_−_ were fixed to a value close to their final value (0.01 and 0.03). This left 4 STDP parameters available for exploration (Figure 6A). In addition, the maximum level of the STDP magnitude ($\bar{\Delta_{\text{w}}}$) was increased from 10 to 20 due to the fact that it appeared to be saturated in the previous genetic search (Figure 4D).

Note that while varying τ_+_ other parameters were allowed to vary to “compensate” for the value of τ_+_. This was a deliberate decision to discover the effect of τ_+_ in the presence of other compensating changes, which is most like the situation *in vivo*.

The results of the search (Figure 6A) show an approximate exponential dependence of the metric η upon the value of τ_+_ (Figure 6B). Higher values of τ_+_ are associated with lower values of the metric η indicating a less realistic anatomical arrangement of synapses. There is also a dependence of the value of *W*_*max*_ upon the value of τ_+_. Apparently, smaller values of τ_+_ led to a compensating effect in the form of a higher “choice” of value for *W*_*max*_.

By examining the final synaptic profile of 100th generation cell models after 10 epochs of learning it can be seen that the longer duration of the positive STDP window has predictable effects (Figure 6C). The longer window associated with higher value of τ_+_ lead to less selectivity of synaptic weights than a cell model with the lower value of τ_+_ (Figure 6C). This lack of selectivity was not completely compensated by any other parameter in the parameter search. It might be thought that a lower value of $\bar{\Delta_{\text{w+}}}$, for example would provide increased selectivity; however, this was not reflected in the genetic search. This was likely due to the fact that the temporal width of the STDP window would not be modified by such a parameter change.

## Discussion

This study demonstrates that it is possible for a spike-timing based synaptic plasticity mechanism to select synapses such that their dendritic delays compensate for input asynchrony. This was shown in a realistic model of the auditory nerve's synapses onto octopus cells in the ventral cochlear nucleus. Parameters associated with a standard STDP window were selected to allow the STDP to guide the selection of the correct subset of synapses. Parameter selection of the STDP learning process was achieved with a simple genetic search. The fitness function for this search was developed to be maximum when the synaptic configuration matched that known to occur in the cochlear nucleus.

Previously published experimental studies on the arrangement of synaptic connections from ANFs to octopus cells include detailed descriptions of their anatomical structures (Oertel et al., 2000; McGinley et al., 2012). Specifically, the experimental observations showed that octopus cell dendrites lie perpendicular to the tonotopically ordered ANFs such that octopus cell dendritic delay compensates for the traveling wave delay. It has been implied that this perpendicular anatomical arrangement is sufficient to provide for the traveling wave compensation, without invoking a need for activity-dependent plasticity. This may be because activity-dependent plasticity, such as STDP, has been less well explored in the auditory brainstem, where cells respond rapidly to stimuli and with very high firing rates. However, activity-dependent plasticity is known to occur throughout the brain and this paper provides a proof-of-principle of how this mechanism may operate in the case of octopus cells. Synaptic weights were adjusted in an activity-dependent fashion, resulting in improved matching between dendritic delay and input asynchrony. Consequently, the observed anatomical organization may arise not only as a result of the previously proposed developmental processes but is also augmented and refined by activity-dependent plasticity.

Evidence suggests that it is dendritic back-propagation or dendritic spikes of the post-synaptic neuron's action potential that provides the learning signal to individual synapses (Golding et al., 2002). This signal would be delayed in its return to the most distal synapses. Given that it appears that the dendritic delay is an important component of the function of octopus cells, further experimental investigation into the influence of back propagation in octopus cells may be valuable. In particular, perhaps the dendritic delay is a constant for small amplitude PSPs, but after an action potential, nonlinear dendritic spikes may lead to faster propagation speeds. For recent work on nonlinear dendritic processing see for example Xu et al. (2012).

This investigation hypothesizes that developmental plasticity can provide synaptic tuning that allows for the detection of simulated clicks. However, there is also evidence that plasticity is maintained in the auditory brainstem of adult maternal mice (Miranda et al., 2014). If this is the case for the circuit of octopus cells with the auditory nerve then this may allow re-tuning, perhaps providing for changes in the traveling wave delay with age, or re-tuning if particular ANFs are no longer functional.

In the present investigation it was found that STDP windows were required to be very brief. It has been shown that, in theory, learning is optimum when the time constant τ for the STDP window matched the time constant τ for the LIF synapse (Kennedy et al., 2014). Cortical neurons investigated in previous experimental work show long STDP windows roughly corresponding to their longer membrane time constants (Markram et al., 1997). In the present model, it is possible that this optimum is not selected by the genetic algorithm due to the unique spike-initiation mechanism of the octopus cell, which is dependent not on the value of V, but on the value of *dV*/*dt* (Ferragamo and Oertel, 2002). This is a topic for future investigation. Future experimental work should aim to discover whether STDP windows much shorter than previously found do exist in the auditory brainstem where membrane time-constants are also much faster.

The number of synapses initially provided to the cell is important. In particular, if there are too few then there will be insufficient numbers of synapses for the final selected subset. In this investigation we chose to provide 1,200 synapses from 400 different ANFs. A more thorough analysis of the exact numbers may be a useful target of future work and may reveal a minimum number beyond which convergence is not possible.

The results suggest that a large number of synapses will be silent during selection using STDP. However, it is possible that these synapses are present only during a critical period after which structural plasticity would lead to their removal.

Like the theory of evolution through natural selection, STDP is a simple rule that can create complex and sophisticated results adapted to local circumstances. While evolution works at the level of the gene (Dawkins, 1976) and time scales of generations of individuals, Hebbian rules work at the level of synapses and over the lifetime of a single individual. In the early investigations into natural selection Charles Darwin collected a multitude of examples of the function of evolution under different circumstances (Darwin, 1859). Perhaps the exploration of STDP and more sophisticated Hebbian rules will undergo a similar history. If that is the case, then the result in this investigation is a small contribution to that process and adds to the number of systems that can potentially be explained using this powerful hypothesis.

## Author contributions

MS undertook the computational work and preparation of the manuscript. All authors contributed to the development of the hypothesis and the analysis of the data.

### Conflict of interest statement

The authors declare that the research was conducted in the absence of any commercial or financial relationships that could be construed as a potential conflict of interest.
